# Comparative and Functional Genomics

**DOI:** 10.1002/(SICI)1097-0061(200004)17:1<VII::AID-YEA14>3.0.CO;2-B

**Published:** 2000

**Authors:** S. G. Oliver


**Yeast**
*Yeast* 2000; **17**: vii.

### Editorial

### *Comparative and Functional Genomics*

The yeast *Saccharomyces cerevisiae* was the first organism from which a chromosome was sequenced and the first eukaryote to have its genome completely sequenced. As I write, the genome sequence of the fission yeast *Schizosaccharomyces pombe* is being completed and this will probably be just the fourth complete eukaryotic genome sequence. *Yeast* (the journal) has played an important role in both the sequencing and post-sequencing phases of genomics through its Sequencing Reports and Functional Analysis Reports. Therefore, it seems appropriate that *Yeast* is acting as 'midwife' to a new journal, *Comparative and Functional Genomics* (*CFG*), that will meet the demands of the post-sequencing era of genomics. However, *CFG* will not deal with just the yeasts and fungi, but with organisms throughout the evolutionary range. Thus, *CFG* has Section Editors for the bacteria, for yeasts, for filamentous fungi, for plants, for *Drosophila*, for *C. elegans*, for the zebra fish, for mouse, and for humans. Moreover, it also has Section Editors to handle all of the major technologies involved in comparative and functional genomics: bioinformatics, transcriptomics, proteomics and metabolomics.

I gave a lecture on functional genomics at a British university recently, where the departmental Chairman, at the end of my talk, remarked that he thought he had 'seen the future, and it's complicated'. I took that to be a comment, not on my limited didactic powers, but on the fact that functional genomics is an open-ended field of study that requires a large number of different approaches. As I have indicated, *CFG* is well equipped to deal with the whole range of approaches and model systems and also, through its parallel electronic publication, is able to present large and complex datasets in an accessible way. However, just as the major challenge to bioinformatics in the post-sequencing era is to extract useful biological knowledge from large and diverse datasets, so any journal dealing with comparative and functional genomics must be able to cut through the complexity and present its readers, whatever their individual research interests, with an accessible and lively view of where the field is going and what new biological insights are emerging. For this reason, *CFG* will present Reviews, Commentaries, Meeting Reports, and Interviews, in addition to Research Articles. Indeed, much of the content of this first issue of *CFG* is material of this type, in order to give you a flavour of the scope and aims of the journal. There is, however, one research paper—from a group in Dublin who played an active role in the yeast genome sequencing project and first recognized that *Saccharomyces cerevisiae* went through a complete genome duplication at some point in its evolutionary history. Their current offering is a genomic sequence comparison between *Fugu* and humans. Thus, their paper nicely symbolizes how *Yeast* (the journal) has given rise to this new, and much more general, publication—*Comparative and Functional Genomics*.

**S. G. Oliver**
Editor-in-Chief

Copyright © 2000 John Wiley & Sons, Ltd.

